# Primary Amelanotic Malignant Melanoma of the Tongue

**DOI:** 10.31486/toj.23.0094

**Published:** 2024

**Authors:** Tarun Kumar, Ruchi Sinha, Amber Parwaiz, Madhu Kumari, Tarique Anwer, Surya Nandan Prasad, Jagjit Kumar Pandey

**Affiliations:** ^1^ Department of Pathology, All India Institute of Medical Science, Patna, Bihar, India; ^2^ Department of Radiodiagnosis, All India Institute of Medical Science, Patna, Bihar, India; ^3^ Department of Surgical Oncology, All India Institute of Medical Science, Patna, Bihar, India

**Keywords:** *Melanoma*, *mouth*, *tongue*

## Abstract

**Background:** Primary malignant melanoma rarely occurs in the oral cavity. The tongue is a particularly unusual primary site; lesions may be pigmented or amelanotic. Primary malignant melanoma is frequently mistaken for squamous cell carcinoma.

**Case Report:** A 27-year-old male presented with a large, painless, ulceroproliferative mass on the dorsal surface of the tongue for 6 months. Squamous cell carcinoma was suspected, and the lesion was biopsied. Histopathology was compatible with primary amelanotic malignant melanoma. The patient had no cutaneous lesions consistent with malignant melanoma, and no definitive metastatic lesions were found. Ultrasound and computed tomography did not reveal any evidence of regional draining lymph node metastasis or suspicious lesions anywhere else in the body. The patient underwent composite resection of the tongue tumor and bilateral neck lymph node dissection, had an uneventful postoperative recovery, but was lost to follow-up.

**Conclusion:** Primary oral amelanotic malignant melanoma is a highly aggressive, potentially fatal tumor and because of its rarity, presents a diagnostic challenge. The ideal treatment modality for primary malignant melanoma of the tongue is poorly defined, but surgery is regarded as the most effective course of therapy.

## INTRODUCTION

In various studies, the incidence of oral mucosal malignant melanoma ranges from 2% to 10% of all melanomas.^1^ The nasal cavity and hard palate are the most frequent sites of occurrence of oronasal melanoma.^2^ Primary tongue malignant melanoma is uncommon, accounting for <2% of all oronasal melanoma.^2^ Mucosal melanomas often display invasive and aggressive clinicopathologic behavior and a dismal prognosis compared to cutaneous melanoma.^3,4^ The 5-year survival rate of oral melanoma is 6.6% to 20%.^5^

The diagnosis of melanoma can typically be made with morphologic investigations when melanin is present, but immunohistochemistry analyses are crucial for amelanotic lesions. Because of the rarity of tongue malignant melanoma, definitive diagnostic tools and therapies have yet to be developed.^3^

We report the case of a 27-year-old male who presented with an ulceroproliferative mass on the dorsum of the tongue. The clinical diagnosis was squamous cell carcinoma, but primary amelanotic malignant melanoma was the final diagnosis on microscopy.

## CASE REPORT

A 27-year-old male presented to the Department of Surgical Oncology with complaints of a painless, nonhealing ulcer of the tongue for 6 months. He had no history of trauma or systemic disease and denied smoking and drinking alcohol. An ulceroproliferative mass measuring approximately 3.5 × 3.2 × 1.5 cm was seen on the dorsum of the tongue. Multiple enlarged lymph nodes were noted over bilateral level 1a, left level 4, and the supraclavicular region. No skin lesions were present on the patient's body, and no definitive metastatic lesions were found. No evidence of excision of melanoma-like lesions or pigmented cutaneous lesions on the body, extremities, head, and neck was seen, and no pigmented lesions in the nasal cavity, pharynx, or larynx were seen.

**Figure 1.**
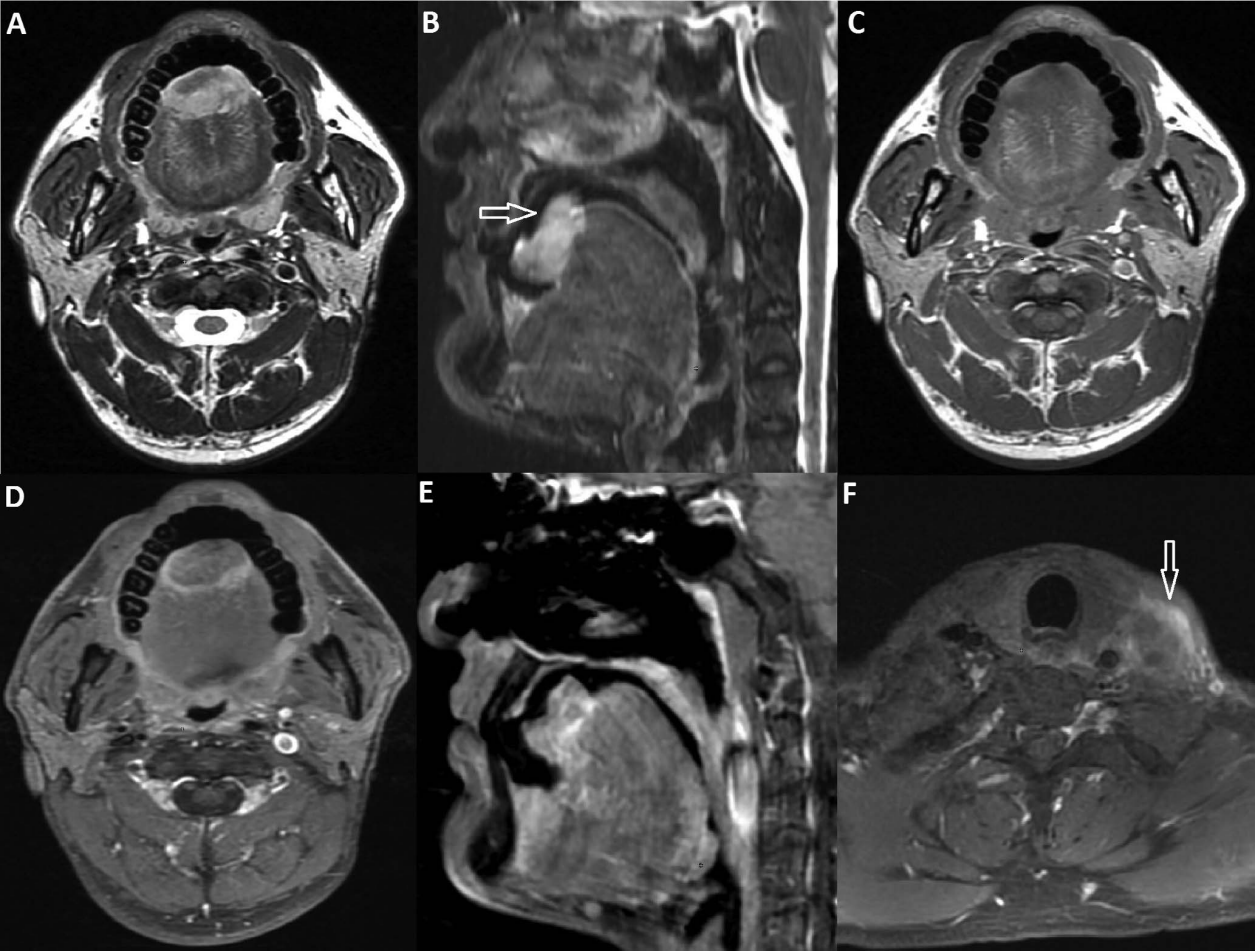
Magnetic resonance imaging (A) T2-weighted axial and (B) short TI inversion recovery sagittal images show a hyperintense, irregular, partly exophytic mass involving the anterior tongue (arrow). (C) On T1-weighted image, the mass is hypointense. Postcontrast T1-weighted (D) axial and (E) sagittal images show heterogeneous contrast enhancement. (F) Postcontrast T1-weighted axial image shows a peripherally enhancing necrotic metastatic lymph node at level 4 on the left side (arrow).

Based on the physical examination, primary squamous cell carcinoma of the tongue was provisionally diagnosed. Punch biopsy of the lesion was taken and reported as a poorly differentiated malignant tumor. Magnetic resonance imaging showed an irregular, exophytic mass involving the dorsum of the tongue and a necrotic metastatic lymph node at level 4 on the left side (Figure 1).

**Figure 2.**
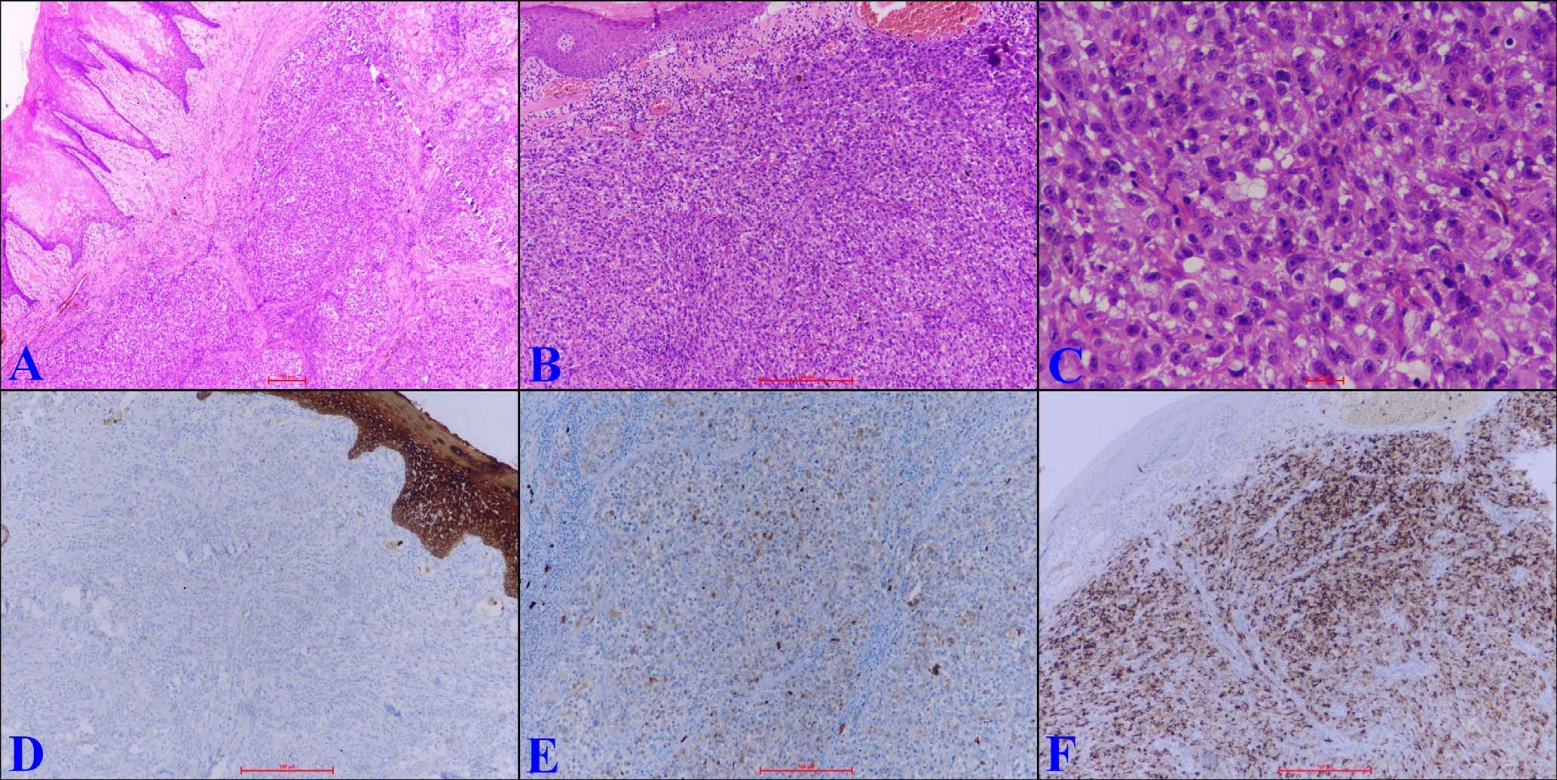
(A) Photomicrograph shows unremarkable overlying stratified squamous epithelium with the presence of a tumor mass in the subepithelium (hematoxylin and eosin [H&E], magnification ×40). (B) The tumor mass is arranged in diffuse sheets composed of epithelioid to spindle cells. No junctional activity was noted (H&E, magnification ×100). (C) The tumor cells display moderate nuclear pleomorphism, large prominent nucleoli, and moderate cytoplasm. Atypical mitosis was noted. Neither pigmentation nor squamous differentiation was seen (H&E, magnification ×400). The tumor cells are (D) immunonegative for pan cytokeratin (immunohistochemistry [IHC], magnification ×100), (E) immunopositive for the S100 protein (IHC, magnification ×100), and (F) immunopositive for HMB-45 (IHC, magnification ×100).

The patient underwent subtotal glossectomy with bilateral neck lymph node dissection. The resected specimen histology revealed an unremarkable overlying epithelium. Subepithelium showed a malignant tumor arranged in diffuse sheets, composed of epithelioid to spindle cells displaying moderate nuclear pleomorphism, large prominent nucleoli, and moderate cytoplasm. A high level of mitotic activity with aberrant mitotic figures was seen. Neither pigmentation nor squamous differentiation was seen in the tumor (Figures 2A, 2B, and 2C). Also, the skeletal muscle underneath was invaded. Neither junctional activity nor epidermal migration was observed. The differential included rhabdomyosarcoma, poorly differentiated squamous cell carcinoma, and amelanotic malignant melanoma. Immunohistochemistry showed positivity for S100 and HMB-45 and negativity for pan cytokeratin (pan CK) and desmin (Figures 2D, 2E, and 2F). Three lymph nodes from the neck dissection showed evidence of metastasis.

The patient had an uneventful postoperative recovery but was lost to follow-up.

## DISCUSSION

Primary malignant melanoma rarely develops on mucosal membranes, and its occurrence in the respiratory, digestive, and urogenital systems is explained by the presence of melanocytes in the mucosal membranes of these tissues.^6^ Although p53 mutations, loss of heterozygosity, and expression of DNA repair protein have been documented, the etiology of mucosal melanoma remains unclear.^7-9^

In a literature review, we retrieved 36 cases of tongue melanoma from 1939 to 2021 in the PubMed and Google Scholar databases, using the keywords “primary melanoma tongue,” “amelanotic melanoma tongue,” “oral melanoma,” and “tongue cancer.”^3-5,10-42^ Baxter (1939) reported the first case of primary malignant melanoma of the tongue.^10^ In the cases retrieved from the literature, the mean patient age was 56.9 years (range, 7 to 90 years), and the highest incidence was seen in the seventh decade (Table).

**Table.** Summary of Studies Reporting Primary Malignant Melanoma of the Tongue

		Clinicopathologic Features of the Tumor				
Study	Patient Age, Years/Sex	Size, cm	Gross Appearance	Site	Lymph Node Metastasis	Melanin
Baxter, 1939^10^	–	–	–	–	–	–
Blackburn, 1951^11^	90/F	–	–	Dorsum	–	–
Moore and Martin, 1955^12^	–/M	–	–	–	–	–
Aponte and Jernstrom, 1956^13^	7/F	–	–	–	–	–
Kragh and Erich, 1960^14^	–	–	–	–	–	–
Amoretti and Oreggia, 1962^15^	75/F	–	–	–	–	–
Milton and Brown, 1965^16^	71/M	–	–	–	–	–
Principato et al, 1965^17^	74/M	3	Fungating mass	Left dorsum	–	Present
Reali-Forster, 1966^18^	–	–	–	–	–	–
Catlin, 1967^19^	–	–	–	–	–	–
Trodahl and Sprague, 1970^20^	69/M	–	–	–	–	–
Kumar et al, 1972^21^	53/M	–	–	Base	–	–
Takagi et al, 1974^22^	–/F	–	–	–	–	–
Lukács, 1980^23^	61/M	–	–	Dorsum	–	–
Kalemeris et al, 1985^24^	47/M	–	–	Dorsum	–	–
Bovo et al, 1996^25^	–	0.4	Black mass	Base	–	Present
Tanaka and Kohama, 1997^26^	62/F	4.7	Black ulcerated mass	Dorsum	–	Present
Spiegel and Singer, 1999^27^	65/F	–	–	Dorsum	–	–
Folz et al, 1998^28^	87/M	–	–	Left base	–	–
Misawa et al, 2000^29^	65/F	5	Nodular irregular mass	Base	–	Present
Chiu et al, 2002^5^	66/F	6.5	Black ulcerated mass	Right side	Absent	Present
Rowland and Schnetler, 2003^41^	53/M	0.8	Pale mass	Dorsum	Absent	Amelanotic
Chikumaru et al, 2008^30^	69/M	1.5	Black pedunculated nodule	Dorsum	Absent	–
Khalifa et al, 2009^31^	73/F	3	Black pigmented ulcerated mass	Dorsum	Absent	Present
Das et al, 2010^32^	13/M	2	Nodular mass	Dorsum	Absent	Amelanotic
Zimmermann et al, 2011^33^	66/M	2.5	–	Base	Absent	Amelanotic
Kumar et al, 2013^34^	50/F	3	Proliferative growth	Dorsum	Absent	Amelanotic
Lee et al, 2013^35^	49/M	NA	Nodular black mass	Dorsum	Absent	Present
Venugopal et al, 2013^4^	19/M	6	Ulcerated mass	Dorsum	Present	Amelanotic
Rubio-Correa et al, 2014^36^	51/M	3	Black pigmented mass	Base	Absent	Present
Alkaff et al, 2017^38^	76/M	4	Exophytic ulcerated mass	Dorsum	Present	Present
Abu-Zaid and Al-Zaher, 2018^37^	30/M	–	Flat to slightly raised purple mass	Dorsum	Absent	–
Swain and Baliarsingh, 2021^3^	68/M	3	Black pedunculated mass	Base, right side	Absent	Present
Leite et al, 2021^39^	62/M	2.5	Solid mass	Tip	Present	Amelanotic
Soares et al, 2021^42^	77/M	–	–	–	Absent	Amelanotic
Motiee-Langroudi et al, 2021^40^	33/F	1	Pigmented ulcerated mass	Dorsum	Absent	Present
Present case, 2023	27/M	3.5	Ulceroproliferative mass	Dorsum	Present (3 of 33 lymph nodes)	Amelanotic

Note: Clinicopathologic data were not reported in many cases, and some cases lacked demographic information. Instances of missing/unavailable data are indicated with a dash (–).

F, female; M, male.

Motiee-Langroudi et al documented that the incidence of oral and tongue melanoma is more frequent in the fourth and seventh decades of life.^40^ Chikumaru et al found no sex difference, while Motiee-Langroudi et al and Chiu et al found that males were more frequently affected than females.^5,30,40^ Ethnic variation has been observed, with the Japanese being particularly more vulnerable to oral malignant melanoma, perhaps related to a genetic or unidentified environmental vulnerability.^5,29,40^ The clinical presentation of tongue malignant melanoma is nonspecific.^3^ Clinical signs typically include nodules or macules at the base of the tongue that are painless and dark brown or black in appearance.^3^ The absence of specific symptoms frequently contributes to a delay in diagnosis; therefore, melanomas of the tongue often manifest in advanced stages before being recognized.^33^ Rowland and Schnetler described tongue malignant melanoma as a pale mass that grew out of a diffuse, faint pigmentation that had previously covered the whole dorsum of the tongue.^41^ Not all mucosal melanomas are pigmented; according to a study by Prasad et al, approximately 38.8% are amelanotic.^43^

In the retrieved studies, tumor size varied, ranging from 0.4 to 6.5 cm, with a mean of 3.08 cm (Table). The dorsal surface is the most common site, followed by the base of the tongue.^4,11,17,23,24,26,27,30-32,34,35,37,38,40,41^ Only Leite et al documented malignant melanoma on the tip of the tongue.^39^ Lymph node metastasis was noted in 4 cases, including the present case.^4,38,39^ Some tongue malignant melanomas are pigmented, while some studies report amelanotic melanoma.^4,32-34,39,41,42^ According to Takagi et al, mucosal melanosis was associated with 66% of oral melanoma: preexisting in 36.2% and concurrent in 29.8%.^22^ The therapeutic strategy and results will differ depending on whether the cancer is a primary oral malignant melanoma or a metastatic deposit from primary skin cancer.^4^ Billings et al observed on histopathology that all metastatic lesions lacked signs of junctional activity in the surrounding mucosa and did not exhibit epidermal migration.^44^ Junctional activity and epidermal migration were present in 44% and 38% of primary lesions, respectively.^44^ Also, extensions of the melanotic pigment into the small salivary glands favored a primary lesion, but these findings were inconsistent.^44^ Hence, we validated the primary malignant melanoma diagnosis by comparing clinical and histologic data. However, a primary malignant melanoma of the tongue can only be diagnosed when all other possible cutaneous or mucosal melanomas have been ruled out.^4^ Except for the absence of pan CK positivity, the immunohistochemical profile of oral malignant melanoma was comparable to that of cutaneous melanoma.^5^ HMB-45 is thought to be more melanoma-specific than the S100 protein.^5,34^ In our patient, the tumor was immunopositive for HMB-45 and the S100 protein, while negative for pan CK and desmin.

For melanoma, surgery is regarded as the most effective course of therapy.^3^ Oral malignant melanoma is difficult to treat because of anatomic constraints, but wide resection with a surgical margin of 2 cm to 5 cm is required for cutaneous melanoma.^3^ The role of radiotherapy is debatable because melanoma is a radioresistant tumor.^5^ In the primary management of unresectable tumors, however, radiotherapy and chemotherapy are crucial for palliation.^5^ Moya-Plana et al recommend neoadjuvant immunotherapy such as anti-PD-1 antibodies nivolumab as the initial step in treatment of head and neck mucosal malignant melanoma.^45^

Patients with oral malignant melanoma typically have a worse prognosis than those with cutaneous tumors.^31^ The poor prognosis may be attributable to various factors, such as the absence of symptoms in the early stages of the disease, the difficulty of performing a wide radical excision because of anatomic restrictions, and a rich blood supply that may promote hematogenous spread.^31^ Recurrence and metastasis are frequent after surgical ablation; most patients die within 2 years.^34^

## CONCLUSION

Although rare, primary malignant melanoma of the tongue might appear as a nonpigmented lesion that resembles squamous cell carcinoma. The head and neck surgeon faces a diagnostic problem when dealing with amelanotic lesions of the oral cavity. A robust index of suspicion and applying a panel of immunohistochemical markers are critical for the correct diagnosis of amelanotic lesions.

## Notes

*This article meets the Accreditation Council for Graduate Medical Education and the American Board of Medical Specialties Maintenance of Certification competencies for Patient Care and Medical Knowledge.*
